# An atypical presentation of infiltrative diffuse low-grade glioma in an adolescent: case report

**DOI:** 10.1186/s12883-025-04259-5

**Published:** 2025-07-01

**Authors:** Zoe Wolfenson, Daniel Benavides, Connor J. Lewis, Gilbert Vezina, Lynne Wolfe, Ellen Macnamara, John Yang, John D. Heiss, Kenneth Aldape, Chris Dampier, Sadhana Jackson, Robert Stone, David Korones, William A. Gahl, Maria T. Acosta

**Affiliations:** 1 Undiagnosed Diseases Program, National Institutes of Health, Bethesda, MD USA; 2 National Human Genome Research Institute, National Institutes of Health, Bethesda, MD USA; 3 Surgical Neurology Branch, National Institute of Neurological Disorders and Stroke, National Institutes of Health, Bethesda, MD USA; 4 Pediatric Oncology Branch, National Cancer Institute, National Institutes of Health, Bethesda, MD USA; 5 Department of Pediatrics, Department of Neurology, University of Rochester, Rochester, NY USA; 6 Department of Pediatrics, Pediatric Palliative Care, University of Rochester, Rochester, NY USA

**Keywords:** Diffuse low-grade glioma, Undiagnosed diseases, Attention deficit hyperactivity disorder, Magnetic resonance imaging

## Abstract

**Background:**

Diffuse low-grade gliomas (dLGG) are rare slow growing brain tumors. Symptoms associated with dLGG typically include seizures, hemiparesis, ataxia, behavioral changes, headaches, and tremors. In this study, we present the case of a thirteen-year-old male admitted to the National Institutes of Health (NIH) Undiagnosed Diseases Program (UDP). To the best of our knowledge, this is one of the only documented cases of an adult-type dLGG diagnosed in a pediatric patient with monitoring of tumor progression for nearly a decade prior to diagnosis.

**Case presentation:**

The patient presented with a history of progressive signal abnormalities on brain magnetic resonance imaging (MRI), refractory to treatment attention deficit hyperactivity disorder (ADHD) and Oppositional Defiant Disorder (ODD), headaches, irritability, and difficulties sleeping. His detailed neurological exam was normal. Following six years of repeated MRI demonstrating increasing infiltrative tumor mass effect throughout gray and white matter, a brain biopsy was performed. The brain biopsy showed white and gray matter with mildly hypercellular areas, and tumor DNA sequencing showed the presence of a canonical *IDH1* mutation. A “watch and wait” approach was adopted resulting from discussions between the patient and his family alongside the medical team with repeated quarterly brain MRI to monitor symptoms and tumor growth.

**Conclusion:**

While behavioral and psychiatric changes are common in brain tumor patients, they typically present alongside neurological symptoms which emphasizes the difficulty in diagnosing cases like this patient’s. Low-grade malignancies should be part of the differential diagnosis in cases with progressive multifocal white matter lesions, despite the absence of the typical neurological focal signs.

## Introduction

Diffuse low-grade gliomas (dLGGs) are rare, slowly growing brain tumors originating from glial cells. The ability of the brain to compensate for slow-growing tumors, oftentimes presenting with minimal clinical signs, makes timely diagnosis difficult [1]. When dLGGs exhibit widespread, typically bilateral infiltration of the brain involving three or more lobes, the presentation may be labeled gliomatosis cerebri [2, 3]. This descriptive terminology is less useful in the context of contemporary World Health Organization (WHO) CNS tumor classifications that emphasize integrated diagnoses summarizing tumors’ morphological, histological, and molecular characteristics [4]. dLGGs may exhibit astrocytic, oligodendroglial, or mixed morphologies. Although the prognosis is generally good in children, it becomes poor when the tumor is multilobar.

Here we highlight a unique presentation of dLGG in a 13-year-old male evaluated by the National Institutes of Health’s (NIH) Undiagnosed Diseases Program (UDP). Our patient presented primarily with intractable attention deficit hyperactivity disorder (ADHD) and Oppositional Defiant Disorder (ODD). His initial magnetic resonance imaging (MRI) findings were incidental, driven by concern for seizures due to a history of seizures in his older brother. Progression of the proband’s brain lesions was closely monitored through serial MRIs, but he remained undiagnosed until age 13 when the MRI findings progressed significantly. Molecular testing of biopsied tissue confirmed the presence of an astrocytoma, IDH-mutant, CNS WHO grade 2. To our knowledge, this is one of the only documented cases of a pediatric patient diagnosed with an adult-type dLGG and monitoring of tumor progression for nearly a decade prior to diagnosis.

## Case report

A 13-year-old male was referred to the NIH UDP by his primary neurologist due to unexplained progressive MRI abnormalities despite extensive evaluations and second opinions from expert centers around the country. The patient was initially evaluated by neurology for difficult-to-treat behavioral issues. At age 3, he began exhibiting inattentive and aggressive behaviors for which he was diagnosed with ADHD and ODD. His behavior became more complex over time despite multiple interventions, and he responded poorly to treatment although his neurological examinations remained entirely normal. His parents reported that he experienced intermittent urinary incontinence that later resolved.

Prenatal history was normal until 28 weeks’ gestation when early labor started. The proband was delivered via normal vaginal delivery at 32 weeks’ gestation weighing 2.13 kg. The infant spent 15 days in the neonatal intensive care unit (NICU) due to apnea, jaundice, bradycardia, hypoglycemia, and feeding difficulties. His family history is positive for cancer, seizures, ADHD, bipolar disorder, heart disease, and strokes.

**Fig. 1:**
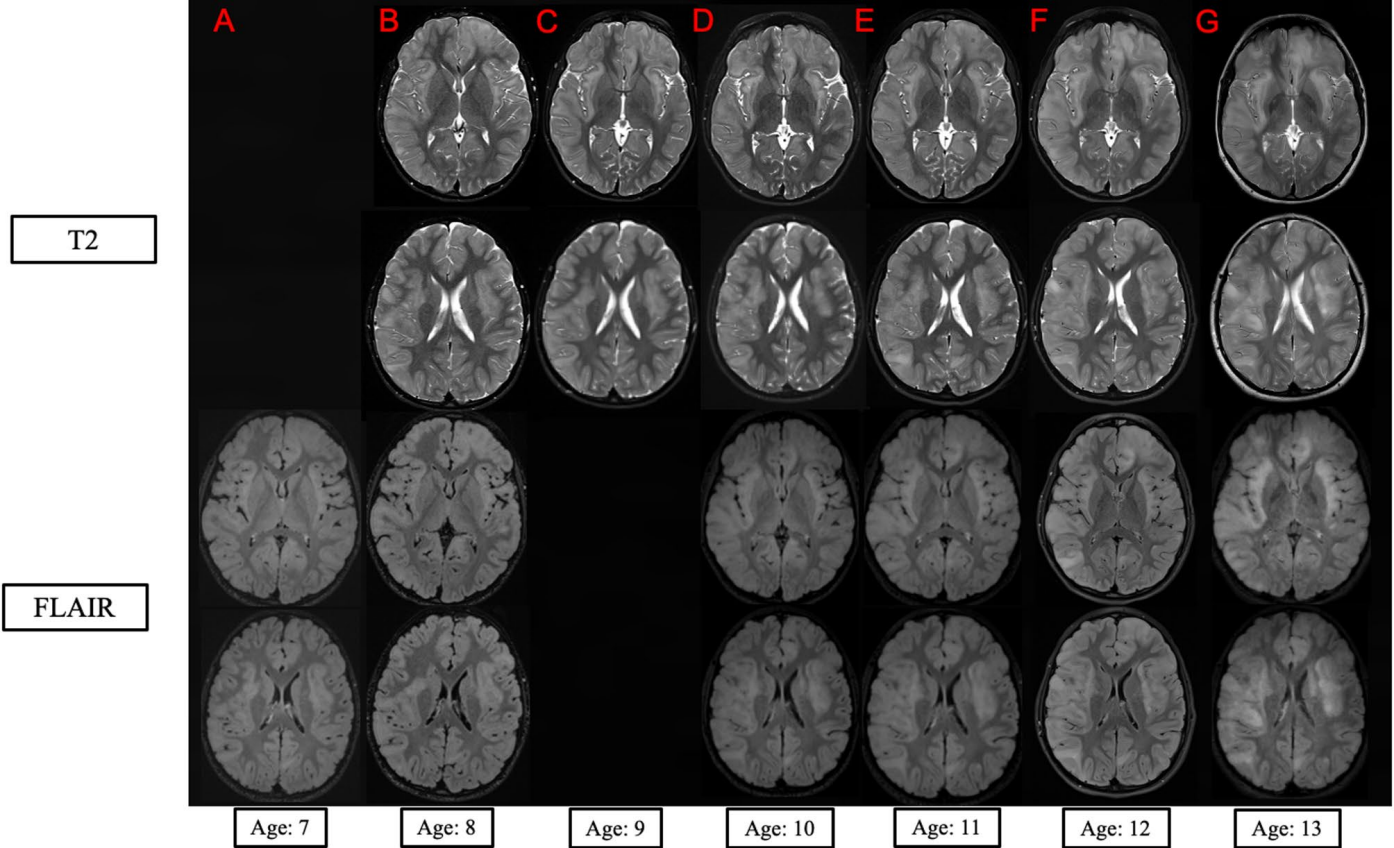
*Imaging*. Axial T2 (top two rows) and FLAIR (bottom two rows) MRI sequences at age 7 (**A**), 8 (**B**), 9 (**C**), 10 (**D**), 11 (**E**), 12 (**F**) and age 13 (**G**) of the patient at the level of insula (top row) and upper lateral ventricles (bottom row), respectively. There is progressive increase in mass effect of the infiltrative tumor throughout the white and gray matter of the insula and both the bilateral frontal and right temporal lobes. There was no Age 7 axial T2 scan, and the Age 9 FLAIR scan was severely motion distorted and not included

At age 4, the parents noted episodes of staring and zoning out. Concern for seizures prompted an Electroencephalogram (EEG), which revealed right frontotemporal epileptiform discharges considered suggestive of benign epileptiform discharges of childhood. The EEG findings had no correlation with the described clinical events, and they resolved spontaneously. As per the focal findings in the EEG, a Brain MRI was completed and identified multiple signal abnormalities throughout the cortex. The patient did not receive antiepileptic medication. A follow-up MRI at age 6 showed progression of these signal abnormalities, which prompted annual MRI evaluations with contrast (Fig. 1). The boy continued being extremely irritable, had difficulty sleeping, and complained about significant headaches that would resolve spontaneously.

At age 8, Brian MRI (Fig. 1B) revealed multifocal asymmetric cortical and subcortical infiltration, with minimal mass effect, most severe in the bilateral insular and sylvian regions, bilateral frontal and the right temporal lobes (Fig. 1B). MRI perfusion showed a symmetrical diffusion pattern with values within the normal ranges at age 10, 11, and 12. Spinal cord MRI were normal at age 7. Ophthalmology evaluation revealed Frisen grade 2–3 papilledema which developed over 5 weeks and was associated with headache and nausea. The diagnosis of pseudotumor cerebri was considered, and the patient responded to treatment with Acetazolamide; within a year, the papilledema resolved and did not recur. Leukodystrophy was the leading diagnosis at this time, however expert evaluation and extensive genetic testing did not identify a genetic cause.

Routine laboratory studies, exome sequencing, and leukocyte lysosomal enzyme studies were unrevealing. No CSF oligoclonal bands or abnormal protein levels were identified.

Following extensive laboratory and clinical evaluation by his home team and by experts at multiple academic hospitals, the patient was referred to NIH UDP at age 13. The T2 weighted and FLAIR contrast MRIs showed a similar distribution of abnormalities but with increasing mass effect (Fig. 1G). The T2 and FLAIR hyperintensities spanned a significant portion of the brain, including the cerebrum, brainstem (including both the medulla and pons), thalamus, and cerebellum. On the T1-weighted scan, the gray and white matter boundary was blurred at throughout both insulae, left frontal pole, and right temporal lobe. The T1-weigthed hypointensities were relatively focal suggesting a higher degree of cellular infiltration in the white matter was present in the right middle temporal gyrus. Spine and the perfusion brain MRI were normal. Dilated optic nerve sheaths suggested increased intracranial pressure, but his detailed neurological exam remained completely normal. Based on the progression of imaging abnormalities and concern for the possibility of an indolent infiltrative glioma, a biopsy was performed.

Open right temporal brain biopsy showed white and gray matter with mildly hypercellular areas composed of diffusely infiltrating neoplastic glial cells with mildly irregular nuclear contours and indistinct cytoplasm in a mildly edematous, fibrillary background. Perineuronal satellitosis was identified. There was no microvascular proliferation, tumor necrosis, or mitotic activity. Immunohistochemical studies showed that neoplastic cells were positive for GFAP, Olig2, and IDH1 p.R132H and negative for synaptophysin, NeuN, and BRAF p.V600E. Nuclear expression of ATRX was lost, and p53 showed strong, positive staining in 5–10% of neoplastic cells. MIB1 highlighted approximately 1% of neoplastic cells. Tumor DNA sequencing confirmed the presence of a canonical *IDH1* mutation (c.395G > A p.R132H, VAF32%) and identified two *TP53* variants (c.747G > T p.R249S, VAF 17%; c.993 + 1G > A, VAF 8.41%). Tumor DNA methylation classification returned a high-confidence match to astrocytoma, IDH-mutant; copy number analysis showed no evidence of loss of the *CDKN2A/B* locus and was negative for 1p19q co-deletion. An integrated diagnosis of astrocytoma, IDH-mutant, CNS WHO grade 2 was given.

Due to the slowly progressive nature of his tumor, the absence of symptoms that would significantly affect his daily life, and the desire to avoid therapy-induced transformation and potential side effects, radiotherapy was not pursued [1, 5–8]. Instead, a “watch and wait” approach was adopted in collaboration with the family, experts, and his local medical team. Quarterly brain MRIs will monitor tumor progression, and interventions will be considered only if symptoms further impact daily activities.

## Discussion

Pediatric dLGG cases are rare, with an annual incidence of 0.1 per million overall and 0.04 per million in children under the age of 14 years [5]. Most patients with dLGGs are diagnosed well into adulthood and experience seizures, hemiparesis, ataxia, behavioral changes, headaches, and tremors [9–11]. For instance, Harrison et al. describe 3 pediatric patients with dLGGs, highlighting the heterogeneity in clinical manifestations including behavioral and psychiatric manifestations prior to objective neurological signs like motor deficits, seizures, or visual field defects [12]. Similarly, Dhakal et al. reports on a case with an initial presentation of cognitive decline prior to seizures and focal signs [13].

These reports of dLGG include behavioral and cognitive changes as part of the clinical disease course. However, the novelty in our case stems from the lack of other neurological manifestations like seizures or focal deficits, despite the long observational period as evident by the serial MRI completed in this case. This unusual presentation is atypical compared with other cases.

The prognosis for pediatric dLGG varies, with significantly longer overall survival than those with high-grade pathology [11]. This survival rate is established by measuring the collective time between diagnosis and death, with diagnosis usually occurring following the onset of significant neurological deficits. However, our patient’s symptoms were mild and atypical relative to the clinical manifestations expected at the time of diagnosis, making an accurate prognosis difficult to establish. 

Here we described a 13-year-old boy with an IDH-mutant astrocytoma who presented primarily with psychiatric symptoms. Despite his profoundly diffuse tumor, his clinical presentation consisted primarily of behavioral manifestations with occasional headaches, nausea, and vomiting, a testament to the plasticity of the brain. His clinical picture was indolent, atypical, and in stark contrast to the overt neurologic deficits usually seen later in the disease course in children with infiltrative dLGGs that include seizures, weakness, sensory loss, language difficulty, visual impairment, and cognitive difficulty [11]. While behavioral changes are common in brain tumor patients, they are usually accompanied by other neurologic symptoms [14–16]. Even with extensive imaging studies and evaluations over nearly a decade, this atypical presentation posed significant diagnostic and therapeutic challenges. Cases like our patient’s, in which behavioral issues are the primary manifestation, are rare and challenging to diagnose, thus emphasizing the importance of considering low-grade malignancy as a potential differential diagnosis for progressive, multifocal white matter lesions. 

While a “wait and watch” approach was adopted for this patient, recent research suggests that course and treatment response may be impacted by the genetic makeup in these patients [17]. Promising new treatments, particularly Vorasidenib, which has shown significant benefits in enhancing progression-free survival and prolonging the time to the next intervention in patients with grade 2 IDH-mutant glioma [18]. This unusual case has provided important insight into the insidious progression of an uncommon brain tumor.

## Data Availability

The data described in this manuscript are available from the corresponding author upon reasonable request.
